# Impact of informal social support on the mental health of older adults

**DOI:** 10.3389/fpubh.2024.1446246

**Published:** 2024-09-26

**Authors:** Yi Dong, Lanyan Cheng, Hailin Cao

**Affiliations:** ^1^School of Public Administration, Hohai University, Nanjing, China; ^2^School of Humanities, Southeast University, Nanjing, China

**Keywords:** mental health of older adults, informal social support, life satisfaction, medical accessibility, social participation

## Abstract

**Objective:**

The role of informal social support in fostering active aging within the context of the “silver wave” is significant. This study investigated how such support influenced the mental health of older adults, with the goal of affirming its indispensable contribution to enhancing their psychological well-being.

**Methods:**

Employing Principal Component Analysis (PCA) to construct the informal social support variable, this study rigorously analyzed the effects and underlying mechanisms of informal social support on mental health in older adults, utilizing data from the 2018 China Longitudinal Aging Social Survey (CLASS).

**Results:**

Informal social support exerted a substantial positive influence on the mental health of older adults, particularly among younger, non-agricultural older adult populations who preferred cohabitation with their children. This form of support significantly enhanced mental well-being by elevating life satisfaction, improving access to medical services, and fostering active social engagement.

**Conclusion:**

This study elucidates the role of informal social support in bolstering the mental health of older adults. Future efforts should focus on fostering a supportive environment that enhances older adult care experiences, reforming the healthcare system to better meet the needs of the aging population, and promoting avenues for social engagement, thereby achieving a balanced integration of their emotional and physical care.

## 1 Introduction

The 2023 United Nations “World Social Report” identified a significant demographic shift, noting that the global population aged 65 and over reached 761 million in 2021, with projections indicating a rise to 1.6 billion by 2050. This demographic transformation will have far-reaching implications for various facets of human activity and societal structure. Consequently, enhancing older adult health and well-being has emerged as a strategic priority for global policymakers. The rapid acceleration of population aging is also associated with increased mental health challenges among older adults (1). The abrupt transition in social roles post-retirement often leaves individuals struggling to adapt, potentially leading to diminished self-worth and psychological issues like loneliness. A World Health Organization report has highlighted that addressing mental health in older adults is integral to fostering active aging. Thus, examining this issue is both a practical response to these mental health challenges in society and an essential component of the broader agenda on active aging.

The mental well-being of older adults has increasingly attracted scholarly attention, emerging as a key aspect in the progression of aging-related initiatives (2). The various determinants influencing older adults’ mental health have stimulated significant public discourse. Current studies indicate a substantial positive correlation between social support and the mental health of older adults, highlighting its substantial impact (3). Social support, a concept rooted in the 1970s, encapsulates the perceived care and assistance received from others. This concept has gained significant traction across disciplines such as Sociology, Psychology, and Medicine. In the context of older adult mental health, researchers have categorized social support into formal and informal types (4). Formal support, typically provided by governmental bodies, institutions, enterprises, and communities, includes mechanisms such as social security systems, medical security frameworks, and policies that prioritize the welfare and dignity of older adults. Conversely, informal support stems primarily from familial connections, neighbors, friends, and peer groups, offering emotional, behavioral, and informational assistance (5). One report has highlighted that the demand for informal older adults support is expected to increase due to larger older cohorts and longer periods of care needs (6). Especially after COVID-19, the informal social support among the older adults is increasing rapidly due to the shortage of medical resources and the cessation or limitation of formal services for older adults (7).

Therefore, this study investigates the influence of informal social support on mental health in older adults by examining the relationship and underlying mechanisms. The research addresses two key questions: (1) To what extent does informal social support affect the mental well-being of older adults? (2) Through what pathways does this influence operate? Utilizing data from the 2018 China Longitudinal Aging Social Survey (CLASS) and applying Principal Component Analysis (PCA), this study provides a comprehensive analysis of the effects and mechanisms by which informal social support impacts the mental health of older adults.

The study makes several distinct contributions. Firstly, it introduced the concept of informal social support, offering a novel lens to assess its influence on mental health in older adults, thereby broadening the existing insights in this area. Secondly, PCA was employed to construct a multidimensional framework for informal social support variables, enhancing the precision of measurement methods and providing empirical evidence at the micro-level for the pathways through which informal social support influences mental health in older adults. Thirdly, the study not only dissected the heterogeneity of informal social support’s role in enhancing mental health in older adults but also rigorously investigated the transmission pathways, focusing on life satisfaction, access to healthcare, and social engagement, thereby extending the scope of current research.

## 2 Literature review

The international society faces growing concerns regarding the mental health of older adult population. Mental health, defined as a state of complete psychological well-being, encompasses positive internal experiences, strong social adaptability, and the effective use of personal potential and social functions. This state is understood as a dynamic and ongoing process of achieving balance (8). Presently, nearly one-third of older adult population experiences psychological disorders (9). When mental health is maintained, older adults tend to lead more joyful lives, which correlates with a heightened sense of subjective well-being (10). Notably, older adult women report higher levels of spiritual well-being compared to their male counterparts (11). Additionally, those who are rural, widowed, or of lower income are at a greater risk of experiencing loneliness (12).

Extensive research has been conducted on factors influencing mental health in older adults. One study emphasized the internal attributes of older adults, noting that enhanced social participation is associated with a deceleration in the progression of depression, ultimately benefiting mental well-being (13). Additionally, cultivating positive lifestyle choices and habits contributes to sustaining mental health in older adults (14). Some researchers have identified a strong correlation between sleep quality, dietary habits, physical activity, and the mental well-being of older adults (15). Further research has highlighted the influence of external factors, noting that, in the context of rapid advancements in information technology, internet usage inversely correlates with depression levels among older adults, while the digital divide poses risks to their mental health (16). Consequently, increased frequency and proficiency in internet usage, along with a broader utilization of its functions, contribute to improved psychological health in this population (17).

Current research has yet to systematically address the effects of informal social support on mental health in older adults. Existing literature, while acknowledging the role of social support, primarily emphasizes its overall impact. For instance, Krause in 2007 found that social support positively correlates with improved mental health and enhances older adults’ sense of life meaning (18). Studies also indicate that informal support networks help alleviate death-related anxiety among older adults, whereas formal interventions, such as participation in major illness insurance and cooperative medical reimbursement, are associated with heightened anxiety about death (19). In addition, informal support exerts a more profound influence on the mental well-being of older adults compared to formal support (20). Wang et al. in 2022 demonstrated that interactions with relatives and friends, facilitated by social media and active engagement, significantly alleviate loneliness among older adults, thereby bolstering their future outlook (21). Li et al. in 2022, examining the role of filial piety within the context of Chinese culture, identified the fulfillment of filial expectations as a critical determinant of life satisfaction in older adults (22). Notably, disharmony within family relationships can extend its impact on the broader aspects of older adult parents’ lives, contributing to reduced overall life satisfaction (23). While some researchers suggest that care from grandchildren can elevate the happiness of older adults, excessive attention may have the opposite effect (24).

The current body of literature offers a substantial basis for this study, yet certain aspects warrant deeper investigation. First, although extensive research has examined mental health in older adults through the lens of social support—both formal and informal—the multitude of indicators complicates the imprecise analysis of their specific impacts on mental well-being. Second, prior studies on informal social support have predominantly employed limited measurement approaches, often focusing solely on interactions with children or relatives. This narrow scope fails to capture a comprehensive, multidimensional framework for understanding the pathways through which informal social support influences mental health.

This study seeks to bridge existing research gaps by first examining the influence of informal social support on mental health in older adults, thereby broadening the scope of inquiry within this field. The analysis specifically incorporated nine indicators drawn from three distinct categories—children, relatives/family members, and friends—to offer a more nuanced and comprehensive understanding of the mechanisms through which informal social support impacts the mental well-being of older adults, supported by robust literature.

## 3 Research design

### 3.1 Data source

The data utilized in this study were sourced from CLASS, a survey primarily designed and implemented through a collaboration between the Development Research Center and the Institute of Gerontology at Renmin University of China, employing Probability Proportional to Size Stratified Sampling (PPS). Initiated in 2014 as a baseline, the survey follows a biennial cycle to systematically capture social and economic background data on China’s older adult population. This longitudinal approach aimed to illuminate the various challenges encountered during the aging process, thereby providing both theoretical and empirical foundations for addressing the implications of China’s aging demographic. In 2018, CLASS expanded its scope to include 28 provinces, municipalities, and autonomous regions, targeting older adult individuals aged 60 and above, with an initial sample size of 11,419. Following data cleaning and the exclusion of missing values, 7,063 valid samples were retained.

### 3.2 Variable selection and processing

The dependent variable in this study was the mental health level of older adults, measured using indicators derived from the CLASS 2018 database. A total of 12 items constituted the relevant indicators (Table 1). Items 1, 4, and 9 corresponded to positive mental health indicators, while the remaining items reflect negative aspects. Responses were scored on a scale of 1 to 3 for “no,” “sometimes,” and “often,” respectively. Negative indicators were reverse-coded to accurately represent the overall mental health level. This variable was continuous, with scores ranging from 9 to 36 points, where a higher score indicated a higher level of mental health.

**Table 1:** Selection of mental health indicators for older adults.

Serial Number	Item	No	Sometimes	Often
1	Were you in a good mood in the last week?	1	2	3
2	Did you feel lonely last week?	1	2	3
3	Did you feel very sad or upset last week?	1	2	3
4	Did you have a great time last week?	1	2	3
5	Did you feel like you did not want to eat last week?	1	2	3
6	Did you have poor sleep last week?	1	2	3
7	Did you think you were useless last week?	1	2	3
8	Did you feel like you had nothing to do last week?	1	2	3
9	Did you find many interesting things in life last week?	1	2	3
10	Did you feel that you were not accompanied last week?	1	2	3
11	Did you feel ignored last week?	1	2	3
12	Did you feel isolated last week?	1	2	3

This study focused on informal social support as the independent variable. PCA was employed to condense indicators such as financial support from children, frequency of contact, household assistance, and communication with family, relatives, and friends, including heart-to-heart conversations and the extent of provided help. Initially, indicators were standardized, and then the reliability of the PCA method is assessed using the Kaiser-Meyer-Olkin (KMO) measure and Bartlett’s test of sphericity. After conducting the analysis in Stata 17, the KMO value was found to be 0.798, and Bartlett’s test of sphericity was significant at *p* = 0, indicating that the PCA results are reliable. Then, the factor matrix was subjected to an orthogonal rotation using the varimax method, yielding the factor loadings and eigenvalues of the principal components. The results are presented in Tables 2, 3.

**Table 2:** Eigenvalues and contribution rates of principal components.

Factor	Eigenvalue	Proportion	Cumulative(%)
PC1	3.6918	0.4102	41.02
PC2	1.5443	0.1716	58.18
PC3	0.9722	0.1080	68.98
PC4	0.8049	0.0894	77.93
PC5	0.5158	0.0573	83.66
PC6	0.4776	0.0531	88.96
PC7	0.4303	0.0478	93.74
PC8	0.2941	0.0327	97.01
PC9	0.2688	0.0299	100.00

**Table 3:** Principal component scoring coefficients.

Index	PC1	PC2	PC3
Financial support from own children	0.0677	0.2404	0.9430
Frequency of contact with own children	0.0700	0.6708	−0.2484
Frequency of household assistance from own children	0.4029	−0.0324	−0.1214
Number of relatives contact	0.4047	−0.0613	−0.1158
Number of relatives chatting heart-to-heart	0.3876	−0.0213	0.0169
Number of providing help by relatives	0.0637	0.6909	−0.0980
Number of friends contact	0.4133	−0.0540	−0.0044
Number of friends chatting heart-to-heart	0.4093	−0.0803	0.0210
Number of providing help by Friends	0.4144	−0.0158	−0.3876

Initially, based on the principle of retaining principal components with eigenvalues greater than 1, two principal components were selected. However, as shown in Table 1, the cumulative contribution rate of these two components is only 58.18%. To retain more information from the original data, the cumulative contribution rate was increased to nearly 70%, leading to the retention of three principal components with eigenvalues of 3.6918, 1.5543, and 0.9722. These components contribute 41.02, 17.16, and 10.80% of the total variance, respectively. To obtain the factor loadings of the principal components, a varimax rotation was applied using the varimax method. Table 3 presents the scoring coefficients for the principal components by Stata 17. The expressions for calculating the principal component scores are [as shown in (Equations 1–3)]:

(1) $$\begin{array}{c} f_{1}=0.0677f_{x1}+0.0700f_{x2}+0.4029f_{x3}+0.4047f_{x4}+0.3876f_{x5}\\ +\;0.0637f_{x6}+0.4133f_{x7}+0.4093f_{x8}+0.4144f_{x9} \end{array}$$

(2) $$\begin{array}{c} f_{2}=0.2404f_{x1}+0.6708f_{x2}-0.0324f_{x3}-0.0613f_{x4}-0.0213f_{x5}\\ +0.6909f_{x6}-0.0540f_{x7}-0.0803f_{x8}-0.0158f_{x9} \end{array}$$

(3) $$\begin{array}{c} f_{3}=0.9430f_{x1}-0.2484f_{x2}-0.1214f_{x3}-0.1158f_{x4}+0.0169f_{x5}\\ -0.0980f_{x6}-0.0044f_{x7}+0.0210f_{x8}-0.3876f_{x9} \end{array}$$

Based on the above expressions, using the variance contribution rates of each component as weights, an evaluation formula for Informal Social Support (ISS) is established [as shown in Equation (4)], which measures the core explanatory variable of informal social support in this study.

(4) $$ISS=\left(41.02f_{1}+17.16f_{2}+10.80f_{3}\right)/68.98$$

Life satisfaction, medical accessibility, and social participation serve as mediating variables in this study. To assess life satisfaction among older adults, the study employed the “satisfaction with current life” item from the CLASS 2018 questionnaire as the primary indicator. Responses were quantified on a five-point ordinal scale, with values ranging from 1 (“very dissatisfied”) to 5 (“very satisfied”). This study operationalized the mediating variable of medical accessibility through three dimensions: health record establishment, free physical examination services, and door-to-door medical services. For health record establishment, the variable was constructed by selecting the database item “whether a health record was established in the past 12 months” and assigning a binary value (1 for “yes” and 0 for “no”). Similarly, the variable for free physical examination services was based on “whether free physical examination services were utilized in the past 12 months,” also coded as 1 for “yes” and 0 for “no.” The door-to-door medical services variable followed the same method, using the item “whether door-to-door medical services were used in the past 12 months” with identical binary coding. Additionally, the variable for older adults social participation was derived from the CLASS2018 database by selecting “whether the individual participated in voting for the local resident committee/village committee in the past 3 years” and assigning a binary value (1 for “yes” and 0 for “no”) to quantify social participation.

This study controlled for variables influencing the mental health of older adults across multiple dimensions, including personal attributes, family characteristics, and socioeconomic status. Specifically, age, gender, marital status, household registration, and health status were accounted for under personal attributes. Family characteristics considered included the number of children, housing type, and land ownership. Socioeconomic status variables included educational level, income, retirement status, and pension location. Internet usage was also controlled, representing the frequency and proficiency of internet engagement among older adults. Additionally, regional variations were addressed through the use of dummy variables. Detailed descriptive statistics were provided in Table 4.

**Table 4:** Descriptive statistics.

VarName	Variable definition	Mean	SD
Explained Variable			
Mental Health	The negative emotions are processed in reverse order, then adding the positive emotions results scores which are range from 9 to 36 points.	27.8039	4.0811
Psychological Consultation	Using psychological counseling services = 1, No = 0.	0.0188	0.1359
Explanatory Variables			
Informal Social Support	Selecting 9 aspects: the child’s financial help, housework support, contact frequency, family or relative support, and friend support and using Principal Component Analysis to obtain a comprehensive score.	3.6100	1.1937
Mediating Variable			
Life Satisfaction	Life satisfaction: very dissatisfied = 1, relatively dissatisfied = 2, average = 3, relatively satisfied = 4, very satisfied = 5.	3.7758	0.8707
Medical Accessibility			
-Health Record Establishment	Establishing health record =1, No = 0.	0.1184	0.3231
-Free Physical Examination Services	Using free physical examination services =1, No = 0.	0.3008	0.4586
-Door-To-Door Medical Services	Using door-to-door medical services =1, No = 0.	0.0316	0.1750
Social Participation	Participated in local residents committee election = 1, no = 0.	0.4096	0.4918
Control Variables			
Gender	Male = 1, No = 0.	0.5024	0.5000
Age	Continuous variable.	71.4515	7.3711
Marital Status	Married = 1, No = 0.	0.6932	0.4612
Education	Educational level.	1.4485	0.7362
Income	Logarithm of income.	8.7025	2.3439
Household Registration	Agriculture = 1, No = 0.	0.5350	0.4988
Health Status	Generate a 1–5 ordinal variable from self-rated very healthy to self-rated very unhealthy.	2.6876	0.8979
Hospitalization Status	Hospitalized in the past 2 years = 1, No = 0.	0.4471	0.9415
Number of Children	continuous variable.	2.6097	1.4272
Network Application	Internet access = 1, No = 0	4.3532	1.4082
Place of Retirement	children’s home = 1, No = 0	1.3413	0.6844
Land Ownership	Land owned = 1, No = 0	0.1878	0.3905
Retirement Status	Retirement = 1, No = 0	0.3647	0.4814
Housing Type	bungalow = 1, building = 0	0.5453	0.4980

## 4 Empirical results and analysis

### 4.1 Empirical test of the impact of informal social support on the mental health of older adults

The baseline regression results, derived using Ordinary Least Squares (OLS), were presented in Table 5. The findings consistently demonstrate that informal support exerted a statistically significant positive effect on the mental health of older adults, maintaining robustness across models with only core explanatory variables and those with all control variables included. For instance, in Model (3), even after incorporating all control variables, informal social support continued to positively influence the mental health of older adults. This suggests that informal support, typically provided by children, relatives, and friends, plays a vital role in enhancing the mental well-being of older adults. Emotional, financial, and caregiving support from core family members significantly influence the mental health of older adults, enhancing their quality of life in later years. Conversely, support from non-core family members, such as friends and neighbors, alleviates the pressures of dependency on children, contributing to improved mental health through role redefinition and identity reinforcement. In the context of an aging society, each incremental increase in informal social support correlates with a 0.402-unit improvement in mental health in older adults. From a human capital perspective, this dynamic holds substantial implications for bolstering national health capital, individual well-being, and overall human capital quality.

**Table 5:** Regression analysis of the influence of informal social support on mental health of older adults.

	Model 1	Model 2	Model 3
Variables	Mental health	Mental health	Mental health
Informal Social Support	0.679***	0.433***	0.402***
	(0.0351)	(0.0396)	(0.0387)
Gender		−0.0652	−0.0667
		(0.0920)	(0.0857)
Age		0.00397	−0.0103
		(0.00746)	(0.00703)
Marital Status		0.714***	0.741***
		(0.107)	(0.0991)
Education		0.276***	0.173***
		(0.0670)	(0.0627)
Income		0.0691***	0.0374
		(0.0226)	(0.0233)
Household Registration		0.956***	0.607***
		(0.135)	(0.125)
Health Status		−0.918***	−0.977***
		(0.0554)	(0.0548)
Hospitalization Status		−0.295***	−0.236***
		(0.0489)	(0.0452)
Number Of Children		−0.0888**	−0.0161
		(0.0386)	(0.0391)
Network Application		−0.353***	−0.204***
		(0.0358)	(0.0365)
Place Of Retirement		−0.345***	−0.454***
		(0.0703)	(0.0657)
Land Ownership		−0.929***	−0.700***
		(0.122)	(0.130)
Retirement Status		0.688***	0.110
		(0.134)	(0.126)
Housing Type		0.0621	0.403***
		(0.101)	(0.101)
Province Effect	NO	NO	YES
Constant	25.38***	28.71***	32.25***
	(0.133)	(0.596)	(0.604)
Observations	9,027	7,063	7,063
R-Squared	0.040	0.154	0.286

Standard errors in parentheses, ***p < 0.01, **p < 0.05, *p < 0.1.

The regression analysis of control variables in Model (3) revealed several key insights. Although age correlated with a gradual decline in mental health among older adults, this relationship lacked statistical significance. Conversely, married older adult individuals exhibited significantly better mental health compared to their unmarried counterparts. Furthermore, the significantly negative coefficients for self-rated health and hospitalization status suggested a direct link between physical and mental well-being, with better physical health corresponding to improved mental health in older adults. Regarding family characteristics, the presence of more children appeared to correlate with a decline in the mental health of older adults, though this relationship did not reach statistical significance. In contrast, socioeconomic status indicators such as income and educational attainment showed significantly positive coefficients, affirming their beneficial effects on mental health in older adults. Moreover, engagement in internet use by older adults was associated with a modest inhibitory effect on their mental well-being.

### 4.2 Robustness test

#### 4.2.1 Instrumental variable method

Previous regression analysis indicated a significant positive impact of informal social support on the mental health of older adults. However, the potential for reverse causality between informal social support and mental health presents a serious endogeneity issue. Emotional or financial assistance from children, family members, and friends can alleviate older adults’ living pressures, mitigate feelings of loneliness and alienation, and enhance mental health levels (25). Conversely, older adult individuals who are ill or widowed, driven by the traditional notion of “raising children to care for oneself in old age,” may place greater demands on their children for financial and emotional support. To mitigate endogeneity issues, including self-selection and omitted variables, this study adopted the approach of Zheng and Zheng in 2017 by selecting the provincial average wage level as an instrumental variable (26). A higher average local salary tends to increase the likelihood that older adult individuals will opt for self-reliance, declining financial or emotional support from children, friends, and others. Additionally, elevated socioeconomic status among older adults correlates with reduced dependence on informal social support (27), thereby meeting the requirements for a valid instrumental variable. However, the correlation between the provincial average wage level and the mental health of older adult individuals proved to be relatively weak, thereby supporting the exogeneity assumption of the instrumental variable. Column (1) of Table 6 presented the results from the Two-Stage Least Squares (2SLS) regression. The analysis confirmed that informal social support continued to exert a significantly positive impact on the mental health of older adults at the 1% significance level, reinforcing the robustness of the findings.

**Table 6:** Robustness testing.

	(1)2SLS	(2) Replace the explained variable	(3) Winsorization treatment
Variables	Mental health	Psychological counseling	Mental health
Informal Social Support	0.880***	−0.0703**	0.433***
	(0.203)	(0.0348)	(0.0431)
Control Variable	Control	Control	Control
			(0.251)
Province Effect	YES	YES	YES
			(0.412)
Constant	30.28***	−2.006***	32.11***
	(0.547)	(0.569)	(0.611)
Observations	7,992	6,330	7,063
R-squared	0.157	/	0.286

Standard errors in parentheses, ***p < 0.01, **p < 0.05, *p < 0.1.

#### 4.2.2 Replacing the explained variable

The main regression employed a composite measure of various psychological emotions to effectively represent the mental health status of older adults. An alternative approach to assess mental health was through the utilization of psychological counseling services, as individuals who opted for these services may exhibited poorer mental health. To ensure robustness, this study incorporated the question “Do you use psychological counseling?” from the CLASS 2018 questionnaire as the dependent variable to further analyze the impact of informal social support on mental health in older adults. As shown in Column (2) of Table 6, even after substituting the dependent variable, the coefficient for informal social support remained significantly positive at the 1% level, confirming that informal social support continued to have a substantial positive effect on the mental health of older adult individuals, and the regression results maintained robustness following this adjustment.

#### 4.2.3 Winsorization treatment

Outlier values in the data could potentially bias the analysis results. To mitigate this, a 5% winsorization was applied to both ends of the distribution of the explanatory variable, reducing the impact of extreme values, followed by a regression analysis. The regression results, presented in Column (3) of Table 6, demonstrated that even after the winsorization treatment, the coefficient for informal social support on mental health in older adults remained significant at the 1% level. This result suggests a robust positive correlation between higher levels of informal social support and improved mental health outcomes in older adults.

## 5 Further analysis

### 5.1 Heterogeneity analysis

The analysis determined that while informal social support generally enhanced the mental health of older adults, this conclusion only reflected the average effect and does not account for the heterogeneous impact across different groups. To derive more nuanced insights, group regressions were conducted based on age, household registration, and retirement location. The detailed results were presented in Table 7.

**Table 7:** Heterogeneity analysis of the impact of informal social support on the mental health of older adults.

	(1) By Age		(2) By Household Registration		(3) By retirement location	
Variables	Low-age	High-age	Agricultural	Non-agricultural	Children	Non-children
Informal Social Support	0.519***	0.396***	0.453***	0.508***	0.457***	0.194**
	(0.0422)	(0.0723)	(0.0536)	(0.0512)	(0.0450)	(0.0775)
Control Variable	Control	Control	Control	Control	Control	Control
Province Effect	YES	YES	YES	YES	YES	YES
Constant	33.66***	32.06***	33.25***	34.21***	32.50***	36.17***
	(0.901)	(1.598)	(0.809)	(0.747)	(0.666)	(1.280)
Observations	5,893	2,099	3,957	4,035	5,337	1,726
R-squared	0.305	0.272	0.294	0.319	0.307	0.270

Standard errors in parentheses, ***p < 0.01, **p < 0.05, *p < 0.1.

#### 5.1.1 Age difference

This study categorized older adults population into two groups—low-age and high-age—based on whether respondents were born before 1943. The regression results in Column (1) of Table 7 revealed that informal social support exerted a more substantial positive effect on the mental health of the low-age older adults compared to their high-age counterparts. A plausible explanation lies in the strong positive correlation between advancing age and the prevalence of physical and mental health challenges. As age increased, support from children, friends, and relatives may have diminishing returns on the health of older adults. High-age individuals are more susceptible to severe life challenges such as disability, cognitive decline, and illness, which can erode their will to live. Consequently, for those with better physical health, informal social support plays a more significant role in enhancing mental well-being, while its impact is less pronounced among those in the high-age category.

#### 5.1.2 Household registration differences

This study investigated the differential impact of informal social support on mental health in older adults based on household registration status, categorizing respondents into agricultural and non-agricultural groups. The regression results presented in Column (2) of Table 7 indicated a stronger positive correlation between informal social support and mental health in the non-agricultural older adult population. In contrast, this effect was comparatively weaker among those with agricultural household registration. Consequently, it is essential for relevant authorities to prioritize the mental health of older adults in agricultural households and improve the effectiveness of informal social support in rural areas.

#### 5.1.3 Place of retirement differences

To examine the influence of retirement location on the effect of informal social support on mental health in older adults, this study utilized the CLASS 2018 survey item, “Where do you plan to retire in the future?” Respondents were categorized into two groups: those planning to retire in their children’s households and those opting for non-children’s households (nursing homes, their own homes, etc.). A group regression analysis was then conducted. The results, presented in Column (3) of Table 7, indicated that informal social support exerted a stronger positive effect on the mental health of older adult individuals planning to retire in their children’s households compared to those choosing other retirement settings. This effect may be attributed to the higher availability of financial and emotional support from children, which enhanced the efficacy of informal social support. Consequently, it is imperative for core family members to consider retirement location choices carefully and prioritize the mental health of older adults.

### 5.2 Analysis of the mechanism of influence

To examine the indirect influence of the mediating variable, this paper draws on the mediation effect model proposed by Wen and Ye (28). Firstly, the impact of informal social support on the mediating variable is tested. Secondly, the influence of the mediating variable on the mental health of the older adults is assessed. Lastly, the combined effect of informal social support and the mediating variable on the mental health of the older adults is examined. The formulas are as follows:

(5) $$\mathrm{ME}D_{i}=\rho_{i}\mathrm{IS}S_{i}+\partial_{i}X_{i}+\vartheta_{i}$$

(6) $$ME_{i}=\omega_{1}\mathrm{IS}S_{i}+\omega_{2}\mathrm{ME}D_{i}+\mu_{i}X_{i}+\pi_{i}$$

In the above equations, $\mathrm{ME}D_{i}$ _irepresents the mediating variable, $\mathrm{IS}S_{i}$ _idenotes informal social support, and $ME_{i}$ indicates the level of mental health in older adults, $X_{i}$ represents the control variables. While $\vartheta_{i}$ and $\pi_{i}$ represent random error terms. If, in Equation 5, $\rho_{i}$ remains significant or decreases slightly, and both $\omega_{1}$ and $\omega_{2}$ are significant in Equation 6, it suggests a partial mediation effect, indicating that the influence of the independent variable on the dependent variable is partly transmitted through the mediating variable. If $\rho_{i}$ becomes insignificant in Equation 5 and both $\omega_{1}$ and $\omega_{2}$ remain significant in Equation 6, this indicates a full mediation effect, meaning the impact of the independent variable on the dependent variable is entirely realized through the mediating variable. If neither $\omega_{1}$ nor $\omega_{2}$ are significant in Equation 6, it implies that no mediation effect exists. The analysis was conducted through three pathways: life satisfaction, medical accessibility, and social participation. The results were presented in Table 8.

**Table 8:** Analysis of the impact mechanism of informal social support on the mental health of older adults.

Variables	(1) First stage					(2) Second stage					Life satisfaction	Medical accessibility			Social participation	Life satisfaction	Medical accessibility			Social participation	Life satisfaction	Health records	Physical examination	Door-to-door medical services	Social elections	Mental health	Mental health	Mental health	Mental health	Mental health
Informal Social Support	0.0310***	0.199***	0.0575***	0.0543*	0.101***	0.370***	0.392***	0.398***	0.399***	0.374***
	(0.0114)	(0.0209)	(0.0156)	(0.0302)	(0.0142)	(0.0383)	(0.0391)	(0.0389)	(0.0387)	(0.0387)
Life Satisfaction						0.807***				
						(0.0598)				
Health Records							0.285*			
							(0.150)			
Physical Examination								0.261**		
								(0.106)		
Door-To-Door Medical Services									0.743**	
									(0.296)	
Social Elections										0.680***
										(0.0951)
Control Variable	Control	Control	Control	Control	Control	Control	Control	Control	Control	Control
Province Effect	YES	YES	YES	YES	YES	YES	YES	YES	YES	YES
Constant	/	−2.486***	−1.571***	−3.070***	1.638***	29.07***	33.72***	33.70***	33.71***	33.02***
	/	(0.305)	(0.235)	(0.462)	(0.214)	(0.641)	(0.581)	(0.580)	(0.580)	(0.588)
Observations	8,497	7,637	8,462	7,835	8,546	7,038	7,063	7,063	7,063	7,063
R-squared	/	/	/	/	/	0.307	0.287	0.287	0.287	0.292

Standard errors in parentheses, ***p < 0.01, **p < 0.05, *p < 0.1.

Table 8, Column (1) presented the first stage of the mediating effect analysis, examining the influence of informal social support on mediating variables. Informal social support markedly enhanced life satisfaction among older adults and exerted a significant positive impact on establishing health records, participating in physical examinations, and utilizing door-to-door medical services, thereby improving medical accessibility for this demographic. Furthermore, it significantly increased their likelihood of engaging in social elections, thereby strengthening their capacity for social participation. The regression results thus suggest that informal social support effectively boosts life satisfaction, medical accessibility, and social engagement among older adults.

Table 8, Column (2), presented the second stage of the mediating effect, analyzing the influence of informal social support and mediating variables on the mental health of older adults. The results indicated that the coefficients for informal social support were significantly positive at the 1% level, as were the coefficients for each mediating variable. Enhancing life satisfaction among older adults contributed to fostering a healthy psychological state. Regarding medical accessibility, measures such as establishing health records, participating in free physical examinations, and utilizing door-to-door medical services significantly elevated the mental health levels of older adults. Additionally, engagement in social elections markedly improved their mental health. Thus, informal social support indirectly enhanced mental health by improving life satisfaction, medical accessibility, and social participation among older adults.

## 6 Discussion

Many older adult individuals are likely to encounter the challenge of shifting social roles following retirement, making attention to their mental health imperative. The decline in cognitive function, coupled with the abrupt lifestyle changes associated with retirement, can lead to psychological difficulties in approximately 85% of older adult population (29). Disengagement Theory posits that societal balance is maintained through a reciprocal interaction between society and the withdrawal of older adults, governed by established rules that remain unaffected by individual desires (30). Consequently, disengagement from social roles and interactions is an inherent aspect of aging, often accompanied by negative emotions. Addressing this requires enhanced psychological support and emotional care from children, relatives, and friends. China’s deep-rooted cultural tradition of respecting, honoring, and caring for older adults has been perpetuated for thousands of years, forming the ethical foundation of the country’s older adults care practices (31). In rural society, cultural norms and beliefs such as “filial piety” and “raising children for old age” play a central role in guiding social behavior. Social expectations further reinforce the obligation for children to provide financial and emotional support to their parents. Consequently, the family-based and home-based pension models have remained predominant due to the historical continuity of these cultural traditions. Additionally, research indicates that older adult individuals with a larger network of relatives and friends are more likely to maintain a positive outlook during the aging process (32). Social support from friends mitigates the risk of chronic diseases that contribute to anxiety in older adults and alleviates symptoms of depression (33). Consequently, informal social support—primarily from children, family, relatives, and friends—emerges as a significant factor positively influencing the mental health of older adults, a key conclusion of this study.

An in-depth analysis was conducted on how informal social support influenced the mental health of older adults. Older adults care extends beyond merely providing basic needs such as support and dependence; it should prioritize enhancing quality of life and overall satisfaction. The predominant Chinese older adults care model, which emphasizes home-based care, effectively improves the quality of life for older adult individuals residing with their children (34). In contrast, those living in older adults care institutions often report lower levels of life satisfaction (35). This correlation between informal social support and life satisfaction has been validated by various studies. Further research indicates that living arrangements play a critical role in determining the life satisfaction levels of older adults (36). Thus, informal social support plays a significant role in enhancing the life satisfaction of older adults, which is a critical indicator of their overall quality of life and mental health (37). Previous studies have identified a negative correlation between poor psychological well-being and life satisfaction among older adults (38), with this relationship being particularly pronounced in cases of depression (39). Additionally, research suggests that self-esteem and family functioning are key factors in boosting life satisfaction among middle-aged and older adult individuals in rural areas (40). This study empirically supported the notion that enhancing life satisfaction was a primary mechanism by which informal social support influences the mental health of older adults.

Beyond life satisfaction, medical accessibility plays a significant role in the mental health of older adults. This study posited that informal social support enhanced access to medical services, thereby influencing mental health in older adults. The financial status of older adults or their families often acts as a key barrier to obtaining social care and professional medical services (41). Scholars have argued that better economic conditions increase the likelihood of choosing social care and professional medical services (42). Consequently, older adult individuals with limited financial resources or low pension incomes who require medical treatment due to illness are more dependent on their children for assistance. Regarding the influence of medical services on mental health, regular physical examinations and effective doctor-patient interactions enhance older adults’ understanding of their health and improve their physical and mental management abilities. In response, some researchers have proposed the “Door-to-door Medical Services” model, which enables older adults to receive follow-up visits, chronic disease management, physical examinations, and other medical services at home. This model fosters a harmonious doctor-patient relationship, as it is rooted in the familiarity of older adults with their local communities, thereby enhancing their sense of identity and comfort (43). Consequently, increased access to medical services, caregiving, and daily companionship are important factors in improving the mental health of older adults.

The study demonstrated that informal social support enhanced the mental health of older adults by encouraging greater social participation. Family support serves as a fundamental catalyst for this participation, making a positive and nurturing family environment essential for bolstering their emotional inclination to engage in social activities. Within the Chinese cultural context, kinship ties, which are central to the Differential Mode of Association, resemble a series of concentric circles. From the kinship networks established through birth and marriage, broader social networks emerge; this pattern extends to geographical relationships as well (44). For older adults in China, the family remains the primary source of support resources (45). Family-based social networks exert a more pronounced influence on the mental health of older adults in China. Women, compared to men, demonstrate a greater willingness and aptitude for cultivating social relationships outside the family, a tendency that becomes increasingly evident with age (46). Consequently, older adult women tend to have broader friendship networks and more extensive overall social connections than their male counterparts (47). Additionally, researchers have highlighted the strong correlation between the health of older adults and their living environment, emphasizing that social integration and interpersonal relationships provide essential meaning to their lives (48). Thus, social participation plays a vital role in enhancing the mental health of older adults.

The findings of this study hold significant policy implications. Addressing the mental health challenges faced by older adults necessitates the cultivation of a supportive environment to improve their care experience, the reform of the medical system to better meet their needs, and the encouragement of social participation to facilitate their continued contributions.

First, to enhance the care experience of older adults, fostering a supportive and harmonious atmosphere is essential. Promoting cultural values such as “filial piety” and “harmony in the family brings prosperity” tailored to local contexts can help establish a peaceful and cohesive family environment. Additionally, influenced by family ties and blood relations, older adults often anticipate spending their later years surrounded by their children and family members. Thus, family-based older adults care models require redefinition, moving beyond rigid adherence to tradition toward fostering relationships grounded in mutual respect and open communication among family members. It is essential to ensure older adults receive full understanding and care while enhancing family cohesion to elevate their life satisfaction and overall happiness.

Second, addressing the needs of older adults requires strengthening the medical system. This involves advancing the reform of medical services, with a focus on enhancing the supply infrastructure and creating an integrated coordination mechanism between comprehensive hospitals and community health organizations. Tailored care services should be provided based on the diverse medical needs of older adults, aiming to establish a comprehensive and effective healthcare system for this population. Moreover, equitable distribution of medical resources is critical, particularly in enhancing policy support for rural and remote regions. Regular physical examinations and psychological care should be provided through government initiatives, ensuring that older adults have consistent access to medical services.

Third, promoting social participation among older adults enables them to remain active contributors to society. Older adult individuals in good health can be encouraged by some public policies to engage in community volunteer services or simple employment within their capabilities. These activities allow them to realize their value and expand their social networks through meaningful societal interaction (49). Additionally, as information technology continues to evolve with older adults-oriented reforms, online participation has emerged as a new avenue for social integration. For those with limited mobility, enhancing digital literacy and bridging the digital divide can expand their opportunities for online social engagement.

## 7 Conclusion

Utilizing data from the 2018 China Longitudinal Aging Social Survey (CLASS), this study employs Principal Component Analysis (PCA) to systematically assess the impact of informal social support on mental health in older adults and its underlying mechanisms. The analysis reveals that informal social support exerts a significant positive effect on mental health, particularly among younger, non-agricultural older adult individuals who prefer cohabitation with their children. Among various forms of informal social support, assistance from family and relatives emerges as the most influential factor in enhancing older adult mental well-being. The mechanisms driving this effect include increased life satisfaction, improved medical accessibility, and greater social participation. This study elucidates the impact of informal social support on enhancing the mental health of older adults. Moving forward, efforts should focus on fostering a supportive environment that enriches the care experience for older adults, reforming the medical system to better address their needs, and encouraging social participation to enable their continued contributions. These measures will help achieve a balanced integration of “spiritual care” and “physical care.”

While this study robustly argues for the relationship between informal social support and mental health in older adults, it still has some limitations as follow. First, this study uses cross-sectional survey data, which has certain limitations in the vertical dimension. Second, this study explored the role of life satisfaction, medical accessibility and social participation as the mediating variables, but there may be multiple mediating pathways from other perspectives. By combining all the analysis, future research could utilize panel data to track individual changes for a better understanding. Additionally, the potential mediating variables within digital and intelligent elements could be further considered to expand the depth and scope of future research in the coming era of artificial intelligence.

## Data Availability

The datasets presented in this study can be found in online repositories. The names of the repository/repositories and accession number(s) can be found at: http://www.isss.pku.edu.cn/cfps/sjzx/gksj/index.htm.
